# Supplementary material for: Trait and Ability Emotional Intelligence and Its Impact on Sports Performance of Athletes
Source: Sports (Basel). 2021 May 10;9(5):60. doi: 10.3390/sports9050060 (PMC8170878; doi:10.3390/sports9050060)
Supplement: Supplementary file 1 [file sports-09-00060-s001.zip › sports-1173264-supplementary.pdf]

TEIQue-SF

Hinweise zum Fragebogen

- Bitte füllen Sie den Fragebogen selbständig und unter ruhigen Bedingungen aus.
- Bitte beantworten Sie jede der folgenden Aussagen, indem Sie jene Zahl einkreisen, die dem Grad Ihrer Zustimmung oder Ablehnung jeweils am besten entspricht. Es gibt keine richtigen oder falschen Antworten.
- Arbeiten Sie zügig und denken Sie nicht zu lange über die exakte Bedeutung der jeweiligen Aussagen nach.
- Versuchen Sie, so genau wie möglich zu antworten.
- Sie haben jeweils sieben Antwortmöglichkeiten, die von 1(stimme absolut nicht zu) bis 7 (stimme absolut zu) reichen.
- Vielen Dank für Ihre Zeit und Ihr Interesse.

1 . . . . 2 . . . . 3 . . . . 4 . . . . 5 . . . . 6 7

Stimme absolut NICHT zu — **Stimme absolut zu**

1. Ich habe kein Problem damit, meine Emotionen in Worte zu fassen	1	2	3	4	5	6	7
2. Es fällt mir oft schwer, Situationen aus dem Blickwinkel einer anderen Person zu betrachten	1	2	3	4	5	6	7
3. Im Großen und Ganzen bin ich eine hoch motivierte Person	1	2	3	4	5	6	7
4. Es fällt mir gewöhnlich schwer, meine Emotionen zu regulieren	1	2	3	4	5	6	7
5. Im Allgemeinen finde ich das Leben nicht besonders erfreulich	1	2	3	4	5	6	7
6. Ich kann gut mit Menschen umgehen	1	2	3	4	5	6	7
7. Ich neige dazu, häufig meine Meinung zu ändern	1	2	3	4	5	6	7
8. Ich kann oft nicht herausfinden, welche Gefühle ich empfinde	1	2	3	4	5	6	7
9. Ich denke, dass ich viele gute Eigenschaften besitze	1	2	3	4	5	6	7
10. Ich finde es oft schwierig, mich für meine Rechte einzusetzen	1	2	3	4	5	6	7
11. Gewöhnlich bin ich in der Lage, die Gefühle anderer Menschen zu beeinflussen	1	2	3	4	5	6	7
12. Im Großen und Ganzen betrachte ich die meisten Dinge eher pessimistisch	1	2	3	4	5	6	7
13. Nahe stehende Personen beschweren sich oft, dass ich sie nicht richtig behandle	1	2	3	4	5	6	7
14. Es fällt mir oft schwer, mein Leben an die gegebenen Umstände anzupassen	1	2	3	4	5	6	7
15. Im Großen und Ganzen kann ich mit Stress umgehen	1	2	3	4	5	6	7
16. Es fällt mir oft schwer, mir nahe stehenden Personen meine Zuneigung zu zeigen	1	2	3	4	5	6	7
17. Normalerweise kann ich mich gut in jemand anderen hineinversetzen und seine / ihre Emotionen wahrnehmen	1	2	3	4	5	6	7
18. Gewöhnlich fällt es mir schwer, meine Motivation aufrechtzuerhalten	1	2	3	4	5	6	7
19. Wenn ich es möchte, finde ich gewöhnlich Mittel und Wege, um meine Emotionen zu kontrollieren	1	2	3	4	5	6	7
20. Im Großen und Ganzen bin ich mit meinem Leben zufrieden	1	2	3	4	5	6	7
21. Ich würde mich selbst als guten Verhandlungspartner / gute Verhandlungspartnerin bezeichnen	1	2	3	4	5	6	7

22. Ich neige dazu, in Angelegenheiten verwickelt zu werden, mit denen ich später lieber nichts mehr zu tun hätte	1	2	3	4	5	6	7
23. Oft mache ich eine Pause und denke über meine Gefühle nach	1	2	3	4	5	6	7
24. Ich glaube, ich habe viele Stärken	1	2	3	4	5	6	7
25. Selbst wenn ich weiß, dass ich Recht habe, neige ich dazu, nachzugeben	1	2	3	4	5	6	7
26. Es scheint, als hätte ich überhaupt keinen Einfluss auf die Gefühle anderer	1	2	3	4	5	6	7
27. Grundsätzlich glaube ich, dass mein Leben recht gut verlaufen wird	1	2	3	4	5	6	7
28. Es fällt mir schwer, gute Beziehungen zu Menschen aufzubauen,sogar wenn diese mir nahe stehen	1	2	3	4	5	6	7
29. Im Allgemeinen kann ich mich neuen Umgebungen anpassen	1	2	3	4	5	6	7
30. Andere bewundern mich für meine Gelassenheit	1	2	3	4	5	6	7

Scoring key: Reverse-score the following items and then sum up all responses

16, 2, 18, 4, 5, 7, 22, 8, 10, 25, 26, 12, 13, 28, 14

*Numbers on the right correspond to the position of the items in the short form of the questionnaire.
**If you would like to derive factor scores based on the long form, see Webnote 2 on the website.

Trait Emotional Intelligence Questionnaire – Short Form (TEIQue-SF). This is a 30-item questionnaire designed to measure *global* trait emotional intelligence (trait EI). It is based on the long form of the TEIQue (Petrides & Furnham, 2003). Two items from each of the 15 subscales of the TEIQue were selected for inclusion, based primarily on their correlations with the corresponding total subscale scores. This procedure was followed in order to ensure adequate internal consistencies and broad coverage of the sampling domain of the construct. Items were responded to on a 7-point Likert scale. The TEIQue has been constructed with the aim of providing comprehensive coverage of the trait EI domain (Petrides & Furnham, 2001).

Petrides, K. V. & Furnham, A. (2001). Trait emotional intelligence: Psychometric investigation with reference to established trait taxonomies. *European Journal of Personality, 15,* 425-448.

Petrides, K. V. & Furnham, A. (2003). Trait emotional intelligence: Behavioural validation in two studies of emotion recognition and reactivity to mood induction. *European Journal of Personality, 17,* 39-57.

Reference for the TEIQue-SF: Petrides, K. V. & Furnham, A. (2006). The role of trait emotional intelligence in a gender-specific model of organizational variables. *Journal of Applied Social Psychology, 36,* 552-569.

Please note that any commercial use of this instrument is strictly prohibited.

If you would like this document in Microsoft WORD, please e-mail me at: k.petrides@ioe.ac.uk.

For more information about the trait emotional intelligence research programme go to: http://www.ioe.ac.uk/schools/phd/kpetrides/trait_ei.htm
